# Disrupting PIAS3-mediated SUMOylation of MLK3 ameliorates poststroke neuronal damage and deficits in cognitive and sensorimotor behaviors

**DOI:** 10.1007/s00018-024-05166-7

**Published:** 2024-03-08

**Authors:** Yu Jiang, Lulu Hu, Baixue Wang, Bingge Zhang, Mengwen Shao, Li Meng, Yan Xu, Rourou Chen, Meng Li, Caiping Du

**Affiliations:** Research Center for Biochemistry and Molecular Biology, Jiangsu Key Laboratory of Brain Disease Bioinformation, Xuzhou Medical University, Xuzhou, 221004 Jiangsu China

**Keywords:** Global cerebral ischemia, Focal cerebral ischemia, Neuroprotection, Hippocampus, Post-translational modification

## Abstract

Activated small ubiquitin-like modifiers (SUMOs) have been implicated in neuropathological processes following ischemic stroke. However, the target proteins of SUMOylation and their contribution to neuronal injury remain to be elucidated. MLK3 (mixed-lineage kinase 3), a member of the mitogen-activated protein kinase kinase kinase (MAPKKK) family, is a critical regulator of neuronal lesions following cerebral ischemia. Here, we found that SUMOylation of MLK3 increases in both global and focal ischemic rodent models and primary neuronal models of oxygen and glucose deprivation (OGD). SUMO1 conjugation at the Lys401 site of MLK3 promoted its activation, stimulated its downstream p38/c-Jun N-terminal kinase (JNK) cascades, and led to cell apoptosis. The interaction of MLK3 with PIAS3, a SUMO ligase, was elevated following ischemia and reperfusion. The PINIT domain of PIAS3 was involved in direct interactions with MLK3. Overexpression of the PINIT domain of PIAS3 disrupted the MLK3-PIAS3 interaction, inhibited SUMOylation of MLK3, suppressed downstream signaling, and reduced cell apoptosis and neurite damage. In rodent ischemic models, the overexpression of the PINIT domain reduced brain lesions and alleviated deficits in learning, memory, and sensorimotor functions. Our findings demonstrate that brain ischemia-induced MLK3 SUMOylation by PIAS3 is a potential target against poststroke neuronal lesions and behavioral impairments.

**Supplementary Information:**

The online version contains supplementary material available at 10.1007/s00018-024-05166-7.

## Introduction

Ischemic stroke causes neuronal damage and leads to high rates of disability and mortality [1]. The main clinical treatments are thrombolysis and thrombectomy to restore blood supply [2, 3]. However, blood reperfusion treatments does not directly intervene in the neuronal damage caused by stroke. Due to the limitations, thrombolysis and thrombectomy are applied to only a minor proportion of stroke patients. Therefore, exploring the molecular mechanisms underlying stroke-induced neuronal injury is essential to identify potential intervention targets.

Mixed lineage kinase 3 (MLK3) belongs to a superfamily of mitogen-activated protein kinase kinase kinase (MAPKKK) and functions as an upstream initiator of multiple signaling events, including the c-Jun N-terminal kinase (JNK) and P38 MAPK pathways, in response to stress, inflammation, and excitotoxic stimuli [4–9]. Upon elicitation, MLK3 is activated by phosphorylation at the Thr277/Ser281 residues [4]. Activated MLK3 then phosphorylates MAPK kinases (MKKs), such as MKK4/7 and MKK3/6, activating the JNK and p38 MAPK pathways. The MLK3–MKK–MAPK signaling cascades contribute to the pathogenesis of ischemic brain lesions [10–12]. MLK3 is a critical upstream regulator of cerebral ischemia, and blocking MLK3 activation exerts neuroprotective effects. Elucidating the regulatory mechanisms underlying MLK3 activation will provide potential therapeutic targets for cerebral ischemic injury.

Protein post-translational modification is critical for regulating protein structure and function. SUMOylation, a regulatory post-translational modification, involves the reversible covalent conjugation of small ubiquitin-like modifiers (SUMOs) to target proteins at lysine residues [13, 14]. Conjugation of SUMO1 with target proteins regulates neuronal functions such as neuronal excitability and synaptic plasticity [15–22]. Mounting evidence indicates that abnormal SUMOylation homeostasis contributes to various neurological disorders, including ischemic stroke, Alzheimer's disease, Huntington's disease, and Parkinson's disease [23–30]. In stroke patients, SUMO1 expression is upregulated in penumbra brain tissues [31]. Moreover, an imbalance in SUMOylation homeostasis has been detected in both global and focal cerebral ischemic animal models [32–40]. However, whether MLK3 is a target of SUMO1 modification and the role of MLK3 SUMOylation in ischemic stroke remains unknown.

SUMOylation is an enzymatic cascade analogous to the ubiquitin pathway, in which the SUMO protein is successively conjugated to target proteins by SUMO-activating enzymes, SUMO-specific conjugating enzymes, and substrate-selective SUMO ligases [14, 23, 24]. The SUMO ligase PIAS3 colocalizes with SUMO1 in the hippocampal neurons [15, 41]. PIAS3 participates in the SUMOylation of various neuronal proteins to regulate neuronal function [15, 18, 21, 22]. However, it is unknown whether PIAS3 is involved in the SUMOylation of MLK3.

In the study, we identified an additional mechanism for MLK3 regulation by SUMOylation, and PIAS3 facilitated MLK3 SUMOylation after cerebral ischemia. Disruption of the MLK3-PIAS3 interaction by the PINIT domain of PIAS3 inhibited MLK3 SUMOylation and its downstream apoptotic signaling and reduced neurological deficits in neuronal and rodent ischemia models.

## Materials and methods

### Antibodies and plasmids

Primary antibodies against PIAS3 (cat #4164S), SUMO1 (4930S), MLK3 (2817S), cleaved caspase-3 (9661S), p38 (9212S), and phospho-JNKs (Thr183 + Tyr185, 4668S) were from Cell Signaling Technology (Danvers, MA, USA). Flag antibody (F1804S) was purchased from Sigma (St. Louis, MO, USA). Myc antibody (05-419) was purchased from Millipore (St. Louis, MO, USA). PIAS3 (sc-14017), JNK (sc-7345), MLK3 (sc-166639), and SUMO1 (sc-5308) antibodies were purchased from Santa Cruz Biotechnology (Dallas, Texas, USA). MAP2 (17490-1-AP) and β-actin (66009-1-Ig) antibodies were purchased from Proteintech (Wuhan, Hubei, China). The phospho-MLK3 antibody (Thr277 + Ser281, AF8079) was purchased from Affinity (Jiangsu, China). Phospho-p38 antibody (ER2001-52) was purchased from HUABIO (Hangzhou, Zhejiang, China). Horseradish peroxidase-labeled goat anti-mouse and anti-rabbit IgG (H + L), Alexa Fluor 488 goat anti-mouse IgG (H + L), and Alexa Fluor 594 goat anti-rabbit IgG (H + L) antibodies were from Thermo Fisher Scientific (Waltham, MA, USA). IRDye®680RD goat anti-rabbit (926–68071) and IRDye®C800CW goat anti-mouse (926–32210) antibodies were purchased from LI-COR Biosciences (Lincoln, Nebraska, USA).

The vector pcDNA3.1( +) was used to express Myc-tagged MLK3, HA-tagged SUMO1, PIAS3, His-tagged wild-type PIAS3 (His-PIAS3 WT), Myc-tagged wild-type PIAS3 (Myc-PIAS3 WT) and its corresponding domain-deleted mutant constructs (AAs 1–500, 1–320, 1–132, and 133–320). The point mutation of MLK3 from lysine 401 to arginine (MLK3 K401R) was generated by QuickChange II XL site-directed mutagenesis kit from Agilent Technologies (Santa Clara, CA, USA).

### Animals

Sprague‒Dawley rats and C57BL/6J mice were group-housed on a 12 h light–dark cycle with free access to food and water before surgery. All experimental procedures were implemented in accordance with the guidelines described in the revised 2011 Chinese Regulations for the Administration of Affairs Concerning Experimental Animals and approved by the Institutional Animal Care and Use Committee of Xuzhou Medical University. All efforts were applied to minimize the potential discomfort and pain of animals during experiments.

### Primary cortical neuron culture and lentivirus infection

Cortical neurons were isolated from the cerebral cortices of E18 rats and plated at a density of 0.8–1.0 × 10^5^ cells/cm^2^ onto poly-D-lysine-coated dishes or coverslips to culture at 37 °C/5% CO_2_ [21, 22]. The culture medium (Neurobasal serum-free medium with 0.5 mM GlutaMax™ and 2% B27) was replaced every 3 days. Lentivirus expressing the PINIT domain of PIAS3 (Lenti-PINIT), negative control, MLK3 WT or its K401R mutant was added to the cortical culture (MOI = 10) at DIV 9.

### Oxygen and glucose deprivation/reoxygenation (OGD/R) treatment

OGD treatment was performed on DIV 13 primary neurons. For OGD treatment, the cortical neurons were kept in DMEM without glucose at 1% O_2_ for 2 h. After reoxygenation for the indicated periods, the neurons were lysed in ice-cold homogenization buffer [20 mM *N*-ethylmaleimide (NEM), 50 mM MOPS (pH 7.4), 320 mM sucrose, 100 mM KCl, 0.5 mM MgCl_2_, phosphatase and protease inhibitors (20 mM β-glycerophosphate, 20 mM sodium pyrophosphate, 50 mM NaF, 1 mM each of EDTA, EGTA, sodium orthovanadate, *p*-nitrophenyl phosphate, PMSF and benzamidine, and 5 μg/ml each of aprotinin, leupeptin and pepstatin A)] and used for immunoblotting and immunoprecipitation.

### Transient global ischemia model

Transient global brain ischemia was induced by the four-vessel occlusion method as described previously [37]. Briefly, male rats (220–300 g) were anesthetized by isoflurane. The vertebral arteries were electrocauterized. On the following day, the carotid arteries were occluded for 15 min to induce ischemia. The sham-operated rats were subjected to the same surgical procedures except for occluding carotid arteries. Rat rectal temperature was maintained at 36.5 °C–37.5 °C during brain ischemia and 2 h of reperfusion.

### Middle cerebral artery occlusion (MCAO) model

The mouse MCAO/R method was used to establish a transient focal cerebral ischemic model [29, 34]. Briefly, male mice (25–30 g) were anesthetized with 2,2,2-tribromoethanol (0.02 mL/g). A 7–0 nylon monofilament with a silicone-embedded front end 200–250 mm in diameter was inserted into the right internal carotid artery (ICA) to block the origin of the right middle cerebral artery (MCA). After 60 min of MCAO, the nylon monofilament was removed to restore the blood (reperfusion). The sham-operated mice underwent the same surgical procedures except for occluding carotid arteries.

### Adeno-associated virus (AAV) injection

The rats (170–180 g) were stereotaxically injected with 7.12 × 10^9^ GC of AAVs expressing the PINIT domain of PIAS3 [AAV-PINIT] or the negative control (AAV-NC) into the bilateral hippocampal CA1 region with two points (relative to bregma: 3.3 mm anteroposterior, 2.0 mm lateral, and 2.8/3.1 mm depth; 3.9 mm anteroposterior, 3.0 mm lateral, and 2.6/3.1 mm depth) and then subjected to a transient global ischemia model after 5 weeks. The mice (20–22 g) were stereotaxically injected with 6.4 × 10^9^ GC of AAV-PINIT or AAV-NC into the right cortex at two points (relative to bregma: 0.8 mm anteroposterior, 2.5 mm lateral, and 2 mm depth;  −1.5 mm anteroposterior, 3.0 mm lateral, and 2 mm depth) and then subjected to the MCAO model after 5 weeks.

### Tissue preparation

Rat hippocampal CA1 or mouse cerebral hemispheres were isolated and homogenized in ice-cold homogenization buffer. The homogenates were centrifuged at 800 × g for 10 min at 4 °C, and the supernatants were collected for immunoprecipitation and immunoblot analysis.

### Immunoblotting

Equal amounts of protein samples were separated by SDS‒PAGE and then electrotransferred to nitrocellulose membranes (Amersham Biosciences). After the membranes were blocked with 3% bovine serum albumin, incubated with the indicated primary antibodies followed by horseradish peroxidase-conjugated secondary antibodies, and then, the membranes were developed with Immobilon® Western Chemiluminescent HRP Substrate (Millipore). The images were obtained and analyzed by the ChemiDoc-It imaging system Fluor Chem FC3 (Protein Sample).

### Immunoprecipitation

Protein samples were diluted with immunoprecipitation (IP) buffer [20 mM NEM, 50 mM HEPES (pH 7.1), 10% glycerol (vol/vol), 150 mM NaCl, 1 mM ZnCl_2_, 1.5 mM MgCl_2_, 1% Triton X-100, 0.5% NP-40, and phosphatase and protease inhibitor as described above] and incubated with the indicated primary antibodies and Protein A + G magnetic beads (Beyotime) overnight at 4 °C. The beads were gathered by a magnetic stand and washed with IP buffer three times. Then, the bound proteins were eluted by boiling for 5 min in Laemmli sample buffer and used for immunoblot analysis.

### Cell culture and plasmid transfection

HEK293T cells or HT22 cells were cultured in DMEM with 10% fetal bovine serum. When the cells reached approximately 90% confluence, plasmids were transiently transfected with polyethylenimine (PEI, Sigma) [21]. Briefly, DNA and PEI were dissolved individually in DMEM, and then, the PEI/DMEM mix was added to the DNA/DMEM mix. After 15 min of incubation, the PEI/DNA/DMEM mixtures were added to the culture medium. A medium change was performed after 2–3 h of incubation. The cells were collected for assays 24 h post-transfection.

### In vitro SUMOylation assay

The SUMOylation kit (Enzo Life Sciences, NY, USA) was used for the in vitro SUMOylation assay. The reaction system [10 × SUMOylation buffer (2 μL), 20 × E1 activating enzyme (1 μL), 20 × E2 conjugating enzyme (1 μL), 20 × Mg-ATP (1 μL), 20 × SUMO1 (WT or mutant, 1 μL), synthesized Myc-MLK3 (390–413) (WT or K401R, 0.4 μg)] was incubated at 37 °C for 4 h [21]. Then, the reaction was boiled in Laemmli sample buffer for 5 min for analysis by immunoblotting.

### His pulldown assay

His-PIAS3 WT or 133–320-Myc was purified with high-affinity Ni-Charge resin (GeneScript, Nanjing, China). Briefly, HEK293T cells overexpressing His-PIAS3 WT or 133–320-Myc were lysed in ice-cold lysis buffer (50 mM NaH_2_PO_4_, 300 mM NaCl, pH 8.0). Cleared lysates were incubated with Ni^2+^-NTA resin. Next, the resins loaded with His-tagged PIAS bait protein were mixed with cell lysate overexpressing MLK3 at 4 °C for 3 h. After 3 washes with wash buffer (50 mM NaH_2_PO_4_, 300 mM NaCl, 60 mM imidazole, pH 8.0), the proteins on the resin were eluted through boiling in Laemmli sample buffer.

### Purification of Tat-tagged PIAS3 (133–320) (Tat-PINIT)

Tat-tagged PIAS3(133–320) cDNAs were first subcloned into the pET-28( +) vector, and then induced to express in the *Escherichia coli* strain BL21. The harvested BL21 cells were resuspended in lysis buffer and then sonicated by 180 cycles of 1-s bursts at high intensity followed by a 3-s cooling period. Then, the lysate was cleared by centrifugation and loaded onto Ni^2+^-NTA resin. The bound proteins were eluted with elution buffer (50 mM NaH_2_PO_4_, 300 mM NaCl, 250 mM imidazole, pH 8.0) and then desalted and concentrated in an Amicon Ultra 10 kD centrifuge tube (Millipore) [21].

### Immunofluorescence

Neurons were first fixed with 4% paraformaldehyde for 20 min at room temperature (RT) and blocked with 10% normal goat serum (NGS) and 0.3% Triton X-100 in PBS for 1 h. Next, the neurons were successively incubated with primary antibodies, Alexa Fluor 594 goat anti-rabbit and Alexa Fluor 488 goat anti-mouse secondary antibodies, and DAPI counterstain. The stained neurons were mounted with ProLong™ Diamond Antifade Mountant (Invitrogen). Confocal images were acquired by a Zeiss LSM710 laser-scanning confocal microscope with 6–8 randomly chosen fields (200 ×) per well. The overall dendritic length and cell numbers were calculated by ImageJ software, and the average dendrite length of a single cell was used for statistical analysis. The data are from a single experiment (four wells) performed in triplicate.

### Propidium iodide (PI) staining

Neurons were first incubated with Hoechst 33342 (0.005 mg/ml) for 10 min and then with propidium iodide (PI) (0.001 mg/ml) for 45 min at 37 °C. Next, the neurons were rinsed with culture medium and imaged with an inverted fluorescence microscope (Olympus) with 6–8 randomly chosen fields (200 ×) per well. The data are from a single experiment (three wells) performed in triplicate.

### Morris water maze

The Morris water maze was used to examine hippocampus-dependent behaviors [29]. In brief, the rats were trained for five consecutive days (4 trials a day). On the sixth day, the hidden platform was removed to administer a probe test for 60 s. The training and test were video recorded and analyzed by ANY-maze software.

### Contextual fear conditioning test

During training, the rats were allowed to freely explore a fear conditioning training box for 2 min. Then, five 2-s foot shocks (0.7 mA; constant current) with a 58-s interval were administered. After 24 h, the rats were placed in the same box for 8 min. The stiffness was recorded and reported as a percentage of the total observation time.

### Modified neurological severity scores (mNSS)

Neurological dysfunction was evaluated by the modified Neurological Severity Score (mNSS) [42]. The mNSS assessment included motor tests (including forelimb flexion, hindlimb flexion, and head deviates, scored 0 to 3), reflexes absent and abnormal movements (scored 0 to 3), beam balance tests (scored 0 to 6), auditory reflex (scored 0 to 1), and corneal reflex (scored 0 to 1).

### TTC staining

The 2-mm mouse brain slices were stained with 1% TTC (2,3,5-triphenyltetrazolium chloride) for 5 min and then fixed with 4% paraformaldehyde. The infarct area of each section was calculated by ImageJ software. The total infarct volume is the sum of the infarct volume of each layer (infarct area × thickness of each layer). The ischemic infarct volume was reported as (volume of contralateral hemisphere−volume of normal tissue of ipsilateral hemisphere)/volume of contralateral hemisphere × 100%.

### Rotarod test

The rotarod test was used to evaluate sensorimotor functions [29]. Briefly, mice were trained on the three days prior to MCAO surgery with three trials per day (5 min training with 30 min internal). The program was set from 5 to 40 rpm/min with 7 rpm/min acceleration. Seven days after MCAO, mice were tested with the same program. The average falling time of three trials was reported.

### Adhesive removal test

In habituation session, each mouse was allowed to explore for 15 min. The left and right paws of the mice were pasted with red circular patches (6-mm diameter) separately. The process of removing the patch was recorded for 2 min. The average time of three trials tearing off the left and right claw patches was used for statistical analysis.

### Statistical analysis

All the data were statistically analyzed by GraphPad Prism 7.0 software. Immunoblot, immunostaining, and PI staining results are presented as the means ± standard deviations (SD), and data for behavioral studies, mNSS, TTC staining and Nissl staining are presented as the means ± standard errors (SE). Comparisons between two groups were performed by paired or unpaired Student’s *t* test. Comparisons among multiple groups in escape latency (Morris water maze test) and mNSS were carried out by two-way ANOVA, while other group comparisons were statistically made using one-way ANOVA followed by Dunnett’s *t* test. *P* < 0.05 was considered significant.

## Results

### MLK3 is modified by SUMO1, and cerebral ischemia increases MLK3 SUMOylation

**Fig. 1:**
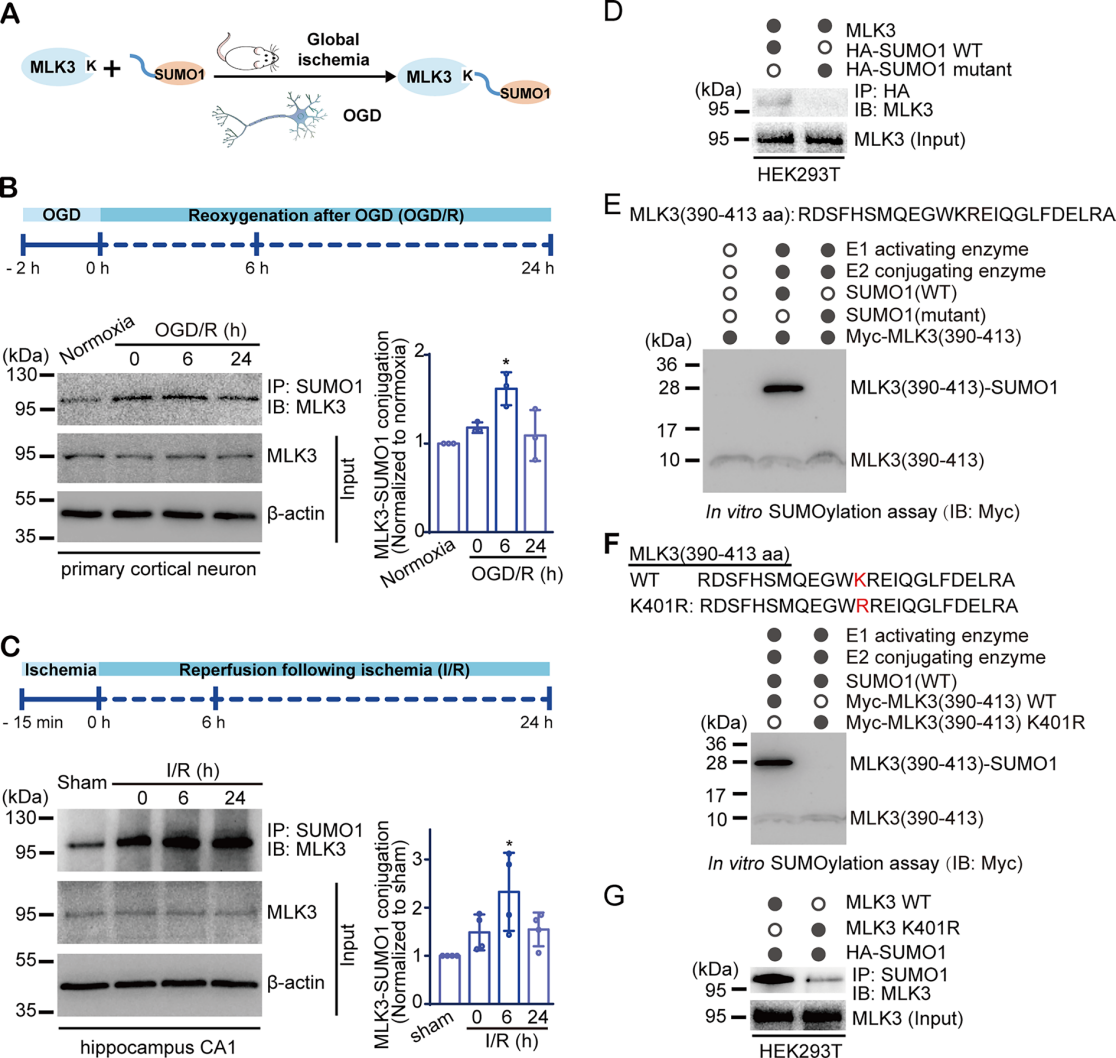
Cerebral ischemia induces MLK3 SUMOylation, and K401 is the major SUMOylation site. **A** Schematic diagram of evaluating MLK3 SUMOylation in OGD and transient global ischemia models. **B**, **C** Time-dependent changes in MLK3 SUMOylation in the primary neuron OGD/R model (B) and in the CA1 region of the rat global I/R model (**C**) by co-IP and immunoblotting. Relative levels were normalized to the normoxia (**B**) or sham (**C**) group. Data are presented as the mean ± SD (*n* = 3–4). *P < 0.05, compared with the normoxia (**B**) or sham (**C**) group; one-way ANOVA with Dunnett’s *t* test. **D** Interaction of MLK3 with SUMO1 in HEK293T cells by co-IP and immunoblotting. **E** In vitro SUMOylation assay for the conjugation of MLK3(390–413) with SUMO1. **F** In vitro SUMOylation assay for SUMOylation of MLK3 at its K401 residue. **G** SUMOylation of full-length MLK3 WT or K401R mutant overexpressed in HEK293T cells

To determine the changes in SUMOylation in cerebral ischemia models, SUMOylation of MLK3 (MLK3-SUMO1 conjugation) was first detected in the normal rat hippocampal CA1 subfield (Figure S1A–S1E). We then measured the conjugation of MLK3 with SUMO1 in OGD and transient global ischemia models (Fig. 1A). In the in vitro rat primary cortical neuron model, MLK3 SUMOylation increased significantly after OGD/R treatment, as shown by coimmunoprecipitation (Fig. 1B). In the in vivo rat global ischemia model (I/R), the level of MLK3-SUMO1 conjugation increased significantly six hours post-I/R in the hippocampal CA1 subfield (Fig. 1C), one of the areas most vulnerable to global ischemia. These data suggest that MLK3 is a novel target of SUMO1 and that brain ischemia promotes MLK3 SUMOylation.

To delineate the conjugation of MLK3 with SUMO1, we first confirmed MLK3-SUMO1 conjugation in HEK293T cells coexpressing MLK3 with wild-type (WT) SUMO1 or an inactive SUMO1 mutant control. As shown in Fig. 1D and S1F–S1G, MLK3 interacted with WT SUMO1 but not with the SUMO1 mutant control. Next, we explored SUMOylation sites in MLK3. Sequence analysis using SUMOplot (http://www.abgent.com/sumoplot), JASSA [43], and GPS-SUMO [44] software predicted that a "WKRE" sequence (amino acids 400–403) of MLK3 was a conserved SUMOylation site, with sequence similarity with the consensus SUMOylation motif of ψKX(E/D) [13, 14] and surface localization in the MLK3 protein (Fig. S2A, B). To validate the predicted SUMOylation site, a Myc-tagged MLK3 peptide representing AAs 390–413 (corresponding to the "WKRE"-containing α-helix) [Myc-MLK3(390–413)] was synthesized chemically for an in vitro SUMOylation assay (Figs. 1E, S2C). A single SUMOylation band at ~ 28 kDa was detected in the presence of SUMO1(WT) but not in the presence of the SUMO1 mutant, indicating covalent conjugation of the MLK3(390–413) peptide with SUMO1. To verify whether K401 in the predicted "WKRE" sequence was the SUMOylation site, a mutated peptide, Myc-MLK3(390–413) K401R, was synthesized chemically for the in vitro SUMOylation assay. As shown in Fig. 1F, SUMOylation of the Myc-MLK3(390–413) peptide was abolished in the K401R mutation group. Moreover, the SUMOylation of full-length MLK3 in cells was significantly diminished by the K401R mutation (Fig. 1G). These findings reveal that the K401 residue was the primary site for MLK3-SUMO1 conjugation, although other SUMOylation sites for MLK3 could not be excluded.

### The SUMO ligase PIAS3 is involved in brain ischemia-induced MLK3 SUMOylation

**Fig. 2:**
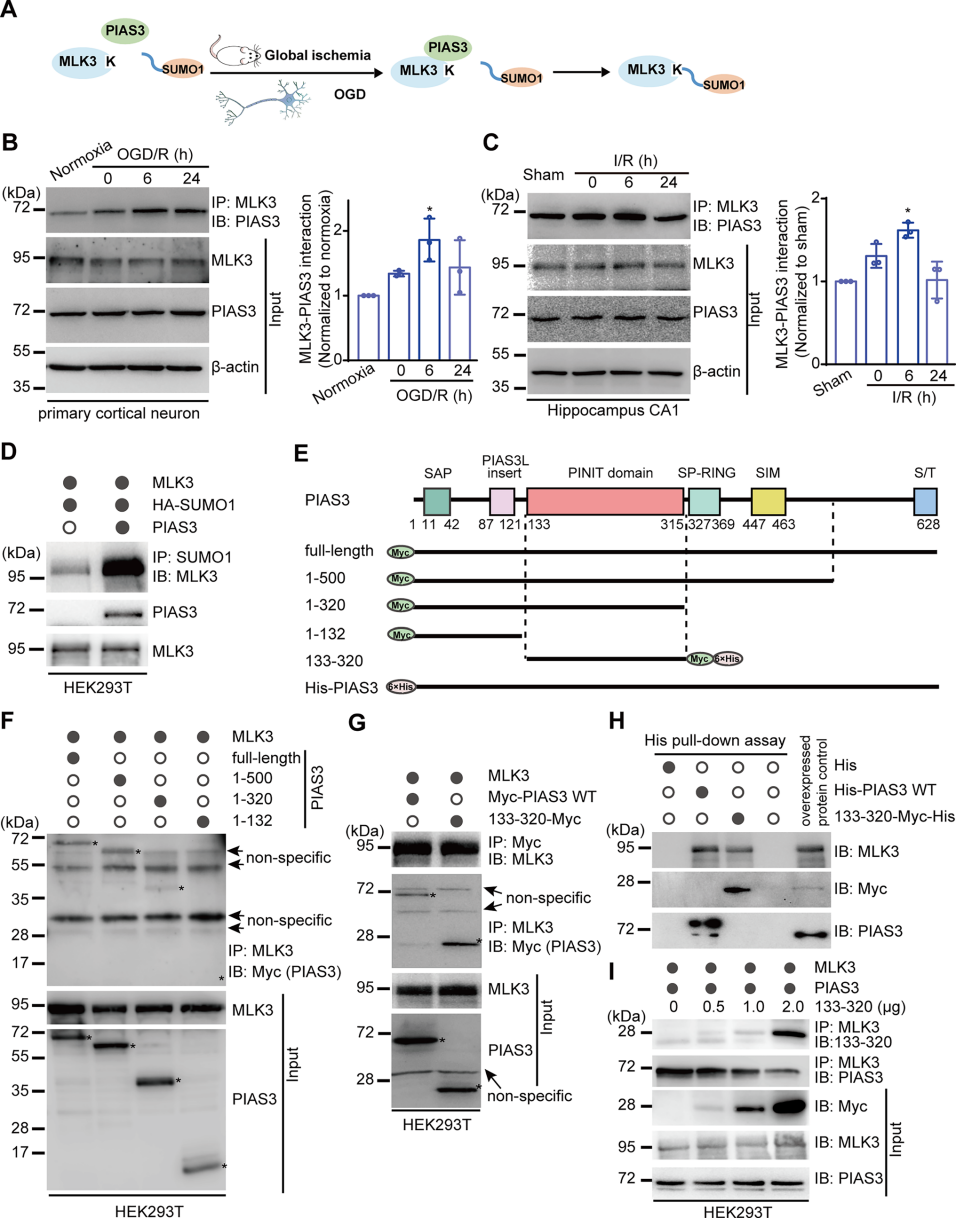
Cerebral ischemia increases the interaction of MLK3 with the SUMO ligase PIAS3, and the PINIT domain of PIAS3 directly binds MLK3. **A** Schematic diagram of assessing the MLK3-PIAS3 interaction in OGD and transient global ischemia models. **B**, **C** Time-dependent changes in the MLK3-PIAS3 interaction in the primary neuron OGD/R model (**B**) and in the CA1 region of the rat global I/R model (**C**) by co-IP and immunoblot analysis. Relative levels were normalized to the normoxia (**B**) or sham (**C**) group. Data are presented as the mean ± SD (*n* = 3). *P < 0.05, compared with the normoxia (**B**) or sham (**C**) group; one-way ANOVA with Dunnett’s *t* test. **D** The role of PIAS3 in MLK3 SUMOylation in HEK293T cells. **E** The structural pattern of Myc-tagged full-length rat PIAS3 (Myc-PIAS3 WT) and its domain-deleted mutants. **F**, **G** The interaction of MLK3 with Myc-PIAS3 WT or its domain-deleted mutants in HEK293T cells. **H** His pulldown assay for the interaction of MLK3 with His-PIAS3 WT or 133–320-Myc-His. **I** Disruption of the MLK3-PIAS3 interaction by 133–320 in HEK293T cells

PIAS3 is a SUMO ligase that participates in the SUMOylation of target proteins to regulate neurological functions [15, 18, 21, 22]. To examine the role of PIAS3 in the SUMOylation of MLK3, the interaction between MLK3 and PIAS3 was first evaluated in a rat primary neuron OGD/R model and transient global ischemia model (Fig. 2A). As shown in Fig. 2B, OGD/R treatment promoted the interaction of MLK3 with PIAS3 in primary cortical neurons, with a time course corresponding to that of MLK3 SUMOylation. Similarly, the interaction between MLK3 and PIAS3 was significantly enhanced in the rat hippocampal CA1 region following I/R (Fig. 2C). To determine whether PIAS3 is involved in the SUMOylation of MLK3, coexpression experiments were performed using HEK293T cells. SUMOylation of MLK3 dramatically increased in the presence of PIAS3 (Fig. 2D).These data suggest that PIAS3 facilitates MLK3 SUMOylation following brain ischemia.

### The PINIT domain of PIAS3 directly binds MLK3

To identify the structural basis for the MLK3-PIAS3 interaction, recombinant constructs coding for Myc-tagged full-length rat PIAS3 (Myc-PIAS3 WT) and its truncated mutants representing AAs 1–500, 1–320, and 1–132 were generated individually (Fig. 2E) and then coexpressed with MLK3 in HEK293T cells. Co-IP experiments showed that interaction with MLK3 was detected in the full-length PIAS3 and its truncated 1–500 and 1–320 groups but not in the 1–132 group (Fig. 2F), suggesting that PIAS3 AAs 133–320, which correspond to the PINIT domain, interact with MLK3. The interaction between MLK3 and the PINIT domain was confirmed using both co-IP (Fig. 2G) and His pulldown assays (Fig. 2H). Moreover, overexpression of the PINIT domain of PIAS3 blocked the MLK3-PIAS3 interaction in a dose-dependent manner (Fig. 2I). These data indicate that MLK3 directly interacts with the PINIT domain of PIAS3 and that overexpression of the PINIT domain disrupts the interaction of MLK3 with PIAS3.

PIAS3 regulates the transcription activity of signal transducer and activator of transcription 3 (STAT3), which has been implicated in cerebral ischemia [45, 46]. In this study, OGD/R-induced STAT3-PIAS3 interaction was not blocked by Tat-PINIT (Fig. S3), which further proves the specific inhibitory effect of Tat-PINIT on MLK3–PIAS3 interaction.

### Disrupting MLK3 SUMOylation with the PINIT domain of PIAS3 reduces neuronal injury in the OGD/R model

Reportedly, MLK3 phosphorylation (activation) peaks at six hours post-I/R, which is consistent with elevated SUMOylation [11, 12]. To elucidate the effects of SUMOylation on the activation of MLK3 and its downstream apoptotic cascades, wild-type MLK3 or its K401R mutant were coexpressed with SUMO1 in HEK293T cells. As shown in Fig. S4, compared to the MLK3 single transfection group, coexpression of MLK3 with SUMO1 facilitated the activation of MLK3 (phosphorylation at T277/S281 sites), increased cleaved caspase-3 levels, increased cell apoptosis, and promoted the phosphorylation (activation) of p38 and JNKs. Moreover, the SUMOylation-deficient K401R mutant had no effect. These results indicate that MLK3 SUMOylation at K401 is vital in regulating its activation and downstream apoptotic signaling.

**Fig. 3:**
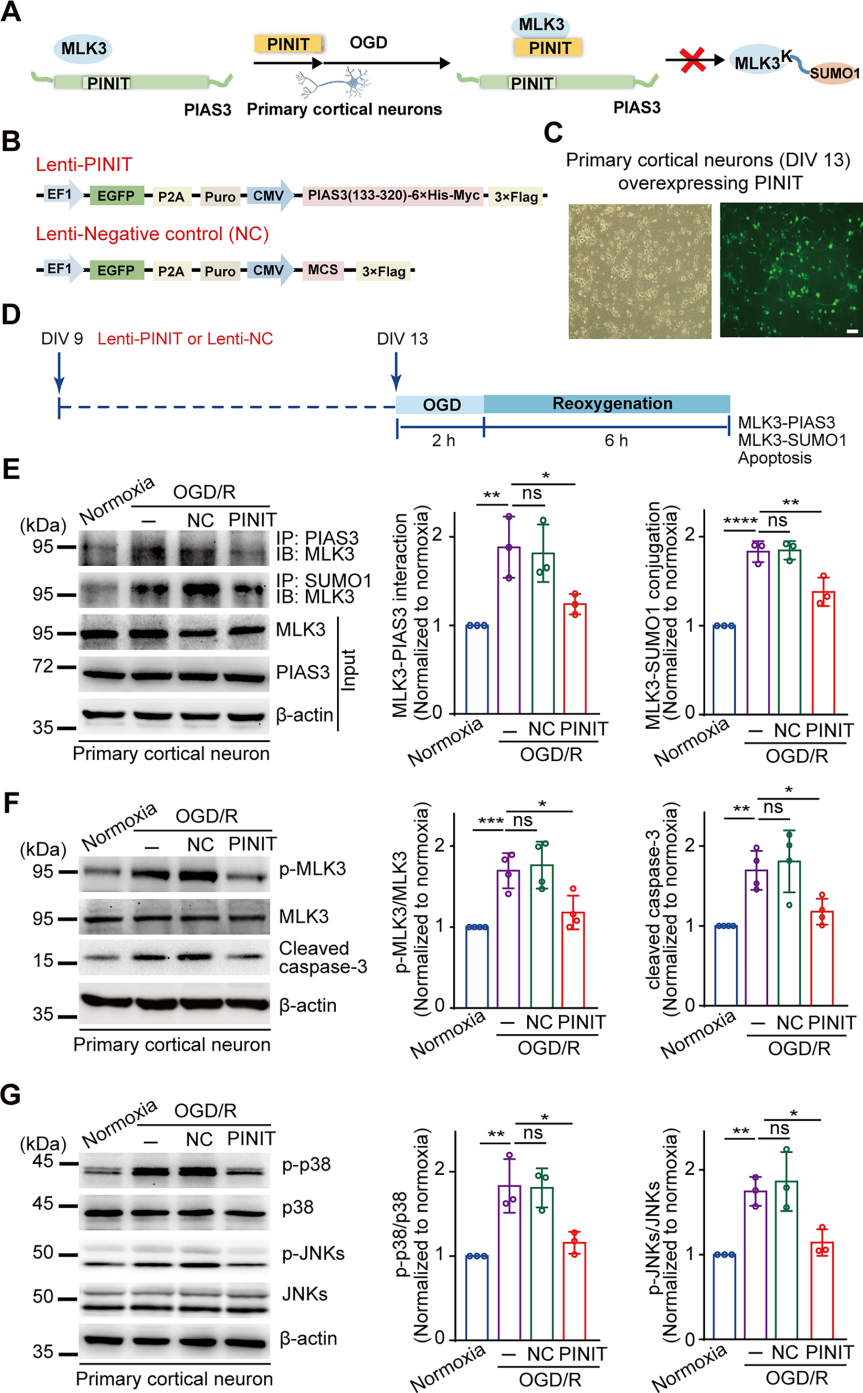
Overexpressing the PINIT domain of PIAS3 blocks the MLK3-PIAS3 interaction and inhibits MLK3 SUMOylation and downstream MLK3-JNK/p38 apoptotic cascade activation after OGD/R. **A** Schematic diagram of blocking PIAS3-mediated MLK3 SUMOylation by the PINIT domain in the OGD model. **B** Diagram of lentiviral plasmids expressing the PINIT domain and negative control. **C** Lentivirus infection efficiency (green) in primary cortical neurons. Bar = 50 μm. **D** Schematic diagram of the overexpression of the PINIT domain in the primary neuron OGD model. **E**–**G** The effects of overexpressing the PINIT domain on the MLK3-PIAS3 interaction, MLK3 SUMOylation (**E**), MLK3 activation/phosphorylation (**F**), and downstream JNK/p38 apoptotic signaling (**G**) after OGD/R. − , without lentivirus infection. Relative levels were normalized to the normoxia group. Data are presented as the mean ± SD (*n* = 3–4). *P < 0.05; **P < 0.01; ***P < 0.001; ****P < 0.0001; *ns* nonsignificant; one-way ANOVA with Dunnett’s *t* test

Next, we explored whether the PINIT domain of PIAS3 interrupts SUMOylation of endogenous MLK3 by PIAS3 in neurons and its effects on neuronal injury in the OGD/R model (Fig. 3A). Primary cortical neurons at DIV 9 were infected with either lenti-PINIT or lenti-NC. At DIV 13, approximately 80% of the neurons expressed EGFP (Fig. 3B–D). As shown in Fig. 3E, overexpression of the PINIT domain blocked the OGD/R-induced MLK3-PIAS3 interaction and SUMOylation of endogenous MLK3 in primary cortical neurons, whereas lenti-NC had no effect. The overexpressed PINIT domain inhibited MLK3, p38, and JNKs phosphorylation (activation) and cleaved caspase-3 expression, which was unaffected by lenti-NC (Fig. 3F–G). These results indicate that overexpression of the PINIT domain intervened in the MLK3–PIAS3 interaction, inhibited MLK3 SUMOylation and enzymatic activity, and ultimately restrained the downstream apoptotic p38/JNKs pathway after OGD/R.

**Fig. 4:**
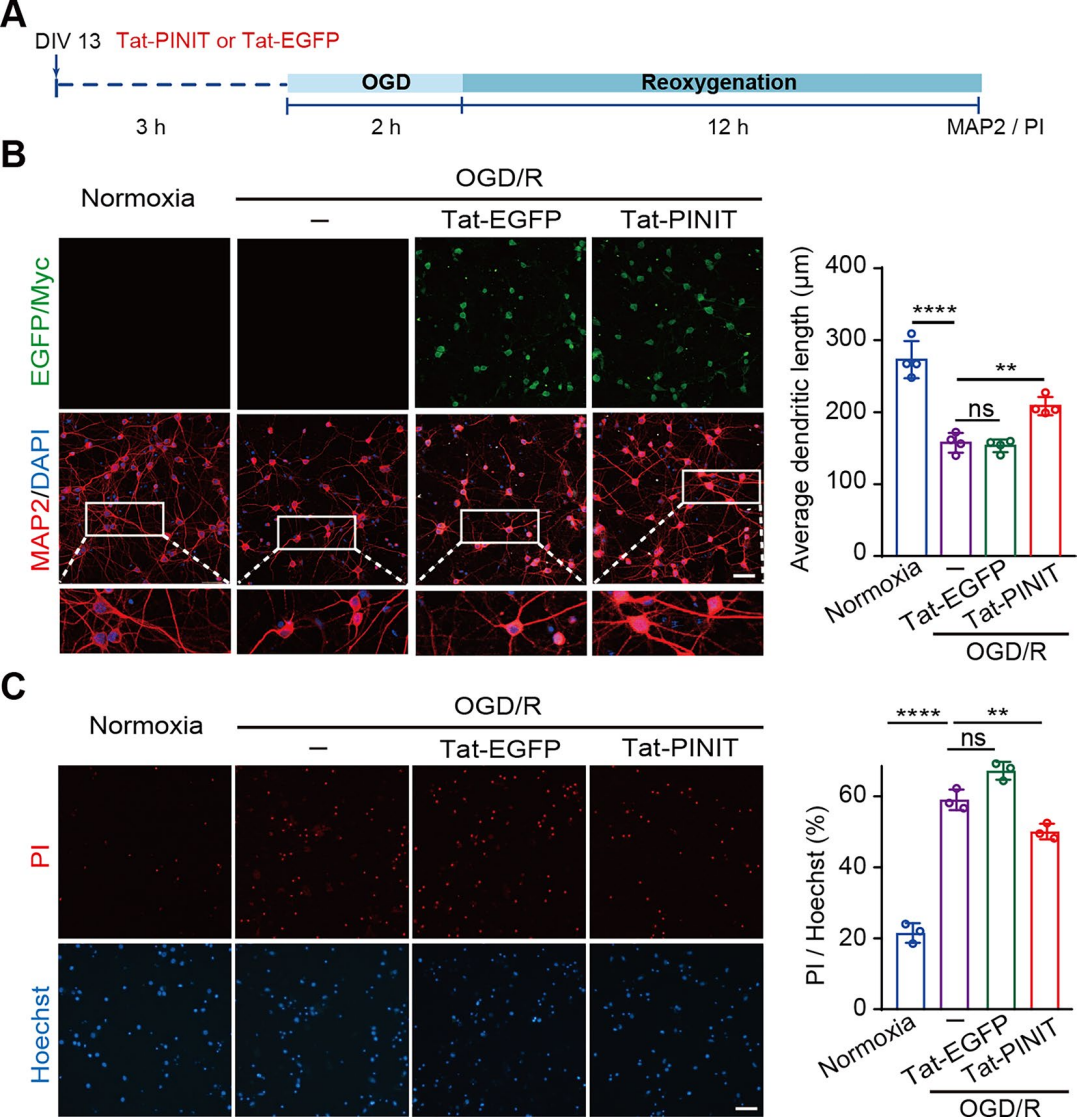
Overexpressing the PINIT domain of PIAS3 alleviates neuronal injury after OGD/R. **A** Schematic diagram of the effect of the penetrating peptide Tat-tagged PINIT (Tat-PINIT) or Tat-EGFP (negative control) on neuronal injury in the primary neuron OGD model. **B**, **C** The effects of overexpressing the PINIT domain (5 μmol/L) on OGD/R-induced neuronal dendrite injury (**B**) and cell apoptosis (**C**). Tat-PINIT was checked by IF with an anti-Myc antibody. The average length of dendrites is presented as the total dendritic length (MAP2) divided by the number of cells. − , without Tat treatment. Bar = 50 μm. Data are presented as the mean ± SD (*n* = 3–4). **P < 0.01; ****P < 0.0001; ns, nonsignificant; one-way ANOVA with Dunnett’s *t* test

To further examine the protective role of the PINIT domain in OGD/R-induced injury, cortical neurons were pretreated with the penetrating peptide Tat-tagged PINIT (Tat-PINIT) or the negative control (Tat-EGFP) for three hours and then subjected to OGD/R treatment (Fig. 4A). As shown in Fig. 4B, the average dendrite length was shortened after OGD/R, and this change was rescued by preincubation with Tat-PINIT but not with Tat-EGFP. Additionally, Tat-PINIT reduced OGD/R-induced apoptosis (Fig. 4C). Moreover, lower level of OGD/R-induced dendrite injury and neuronal apoptosis were observed in the Lenti-MLK3 K401R mutant group compared with the WT group (Fig. S5). Together, these results indicated that Tat-PINIT has neuroprotective effects against OGD/R-induced neuronal damage.

### Interrupting MLK3 SUMOylation with the PINIT domain of PIAS3 reduces neuronal injury and relieves learning and memory defects after I/R

**Fig. 5:**
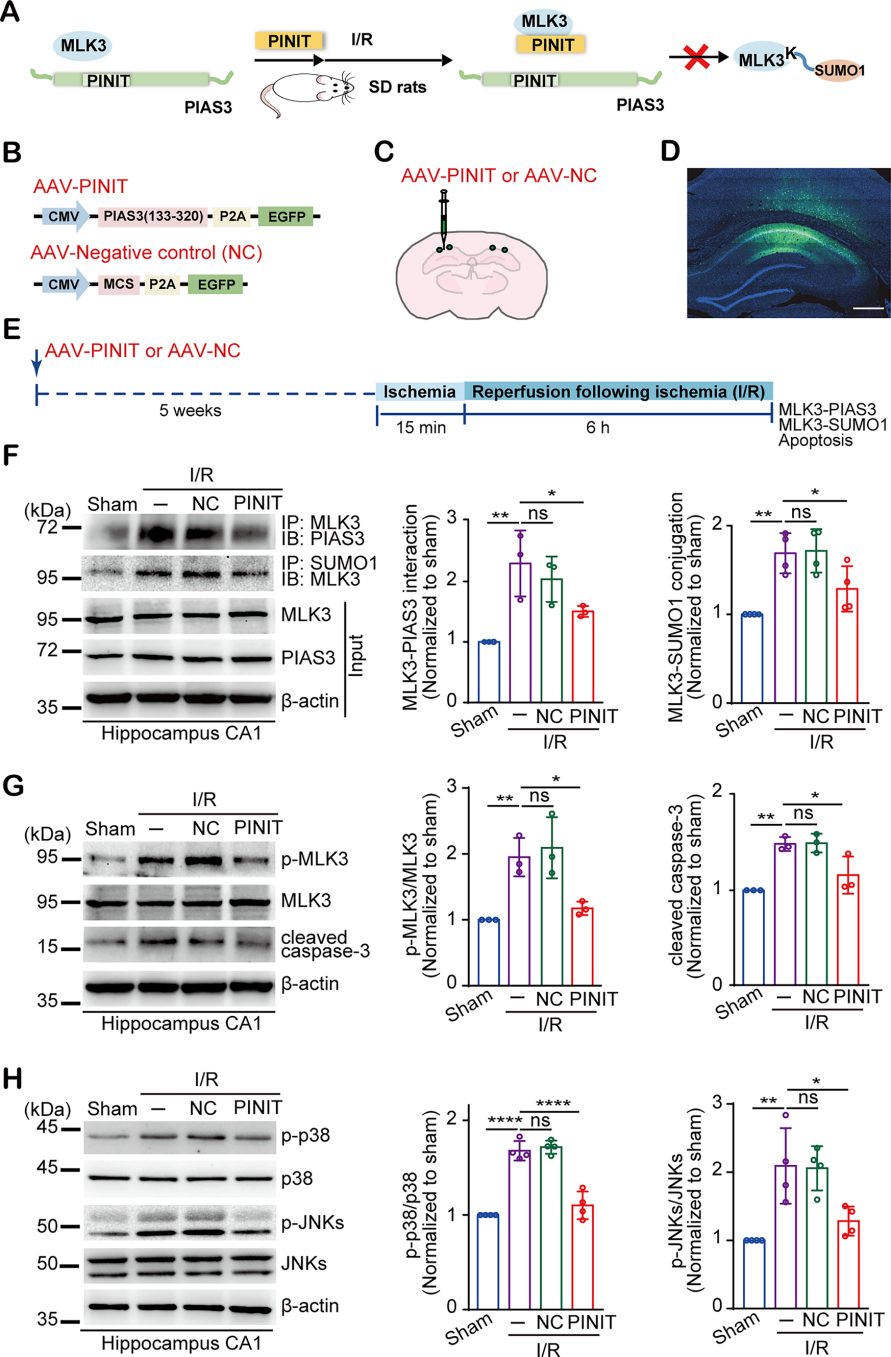
Overexpressing the PINIT domain of PIAS3 inhibits global cerebral ischemia-induced MLK3 SUMOylation and downstream apoptotic cascade stimulation in the hippocampal CA1 subfield. **A** Schematic diagram of the PINIT domain blocking PIAS3-mediated MLK3 SUMOylation in a rat global ischemia model. **B** Diagram of AAVs expressing the PINIT domain and negative control. **C** Schematic representation of AAV injection into the rat bilateral hippocampal CA1 subfield. **D** AAV infection (green) in the hippocampal CA1 region. Bar = 50 μm. **E** Schematic diagram of AAV recombinant overexpression in a rat global ischemia model. **F**–**H** The effects of PINIT overexpression on the MLK3-PIAS3 interaction, MLK3 SUMOylation (**F**), MLK3 activation/phosphorylation (**G**) and downstream apoptotic cascade activation (**H**) after I/R. − , without AAV injection. Relative levels were normalized to those of the sham group. Data are presented as the mean ± SD (*n* = 3–4). *P < 0.05; **P < 0.01; ****P < 0.0001; one-way ANOVA with Dunnett’s *t* test

Next, we evaluated the neuroprotective effect of the PINIT domain of PIAS3 against transient global ischemia-induced neuronal injury. The recombinant AAV encoding the PINIT domain of PIAS3 (AAV-PINIT) and negative control empty vector AAV (AAV-NC) were injected into the bilateral rat hippocampal CA1 region, an area vulnerable to global ischemia, with two points in each hemisphere (Fig. 5A–C). After five weeks, broad expression of AAV-PINIT was observed in the hippocampal CA1 region (Fig. 5D). The rats were subjected to 15 min of ischemia followed by six hours of reperfusion, and the hippocampal CA1 subfield was extracted to assess the MLK3-PIAS3 interaction, MLK3 SUMOylation, and downstream apoptosis signaling (Fig. 5E). As shown in Fig. 5F–H, overexpressing PINIT interrupted the interaction between MLK3 and PIAS3, inhibited MLK3 SUMOylation, reduced the phosphorylation of MLK3, p-38 and JNKs, and decreased cleaved caspase-3 levels. These data reveal that the PINIT domain interferes with the MLK3-PIAS3 interaction, blocks MLK3 SUMOylation and catalytic activity, and decreases downstream p38/JNK apoptotic cascades after I/R.

**Fig. 6:**
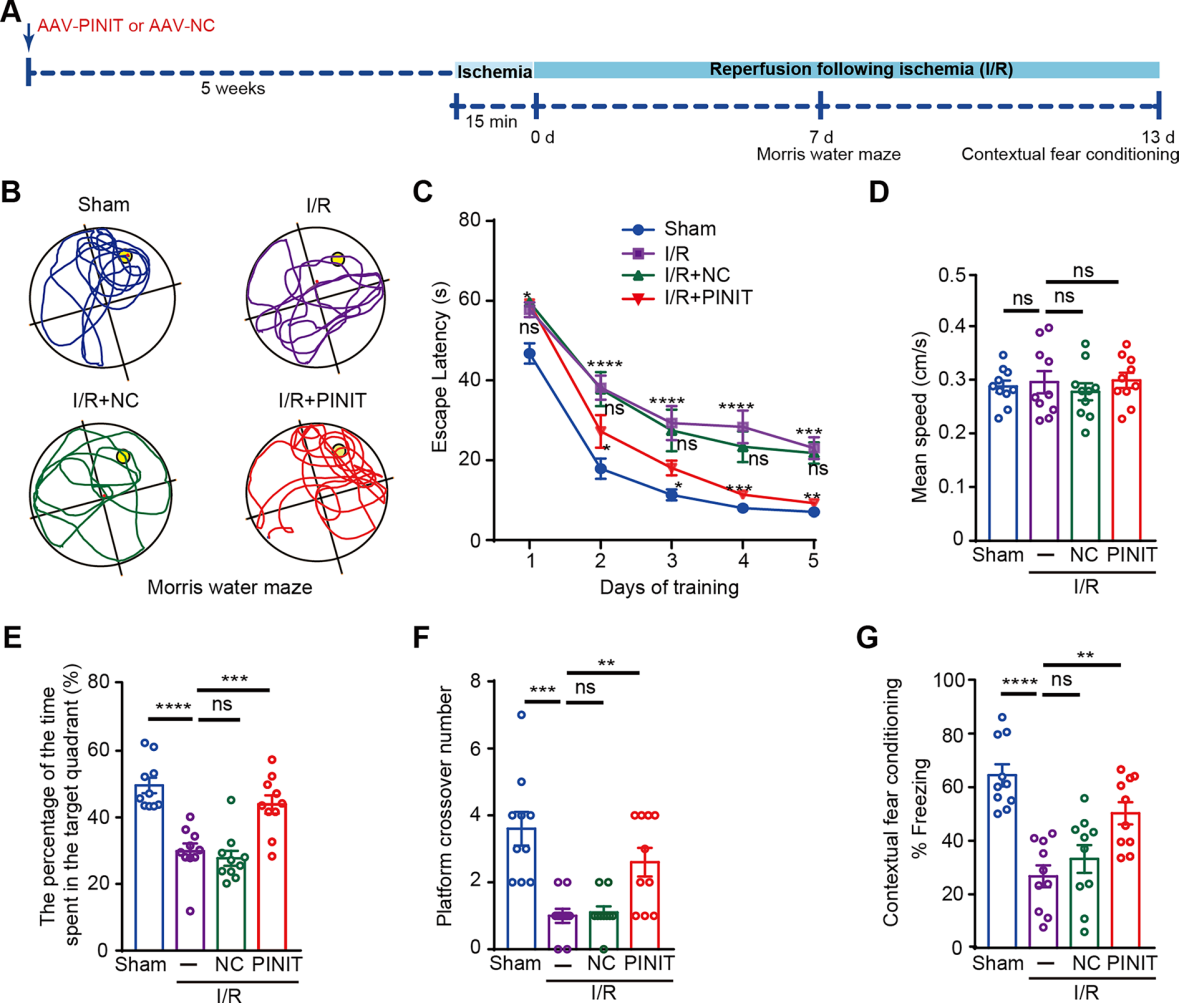
Overexpressing the PINIT domain of PIAS3 relieves learning and memory defects in rats after global cerebral ischemia. **A** Schematic diagram of AAV recombinant overexpression in a rat global ischemia model. **B**–**F** Effects of overexpressing the PINIT domain on spatial memory (Morris water maze) in a rat I/R model. **B** Representative navigation traces on Day 7 in the Morris water maze test. **C** Escape latency over the five-day training session. **D** Mean swimming speed. **E** Percentage of time spent in the target quadrant. **F** Number of platform crossovers. **G** Effects of overexpressing the PINIT domain on contextual fear conditioning in a rat I/R model. Data are presented as the mean ± SEM (*n* = 10). *P < 0.05, **P < 0.01, ***P < 0.001, and ****P < 0.0001; ns, nonsignificant. Two-way ANOVA with Dunnett’s *t* test (**C**), one-way ANOVA with Dunnett’s *t* test (**D**–**G**). − , without AAV injection

The hippocampus CA1 subfield is one of the regions most vulnerable to global ischemia, and is involved in spatial learning and memory. Therefore, we next examined the effects of PINIT overexpression on spatial learning and memory impairments post-brain ischemia. After injection of AAV-PINIT or AAV-NC for five weeks, the rats were subjected to the Morris water maze test and contextual fear conditioning 7 and 13 days post-I/R, respectively (Fig. 6A). During the five days of training in the Morris water maze, the navigation paths were recorded and analyzed (Fig. 6B). I/R rats presented a longer escape latency, which was shortened by treatment with AAV-PINIT but not with AAV-NC (Fig. 6C). On the last day of training, there was no significant difference in the mean swimming speed between the groups (Fig. 6D). Treatment with AAV-PINIT, but not AAV-NC, rescued the I/R-induced reduction in the percentage of time spent in the target quadrant and the number of platform crossovers (Fig. 6E, F). In the contextual fear conditioning test, freezing time was significantly reduced in the I/R-treated rats. AAV-PINIT increased the I/R-induced freezing time, whereas AAV-NC had no effect (Fig. 6G). These results demonstrate that overexpressing the PINIT domain of PIAS3 rescues hippocampus-dependent spatial learning and memory defects in global ischemia.

### Overexpressing the PINIT domain of PIAS3 reduces brain infarction and ameliorates sensorimotor function defects after MCAO/R

**Fig. 7:**
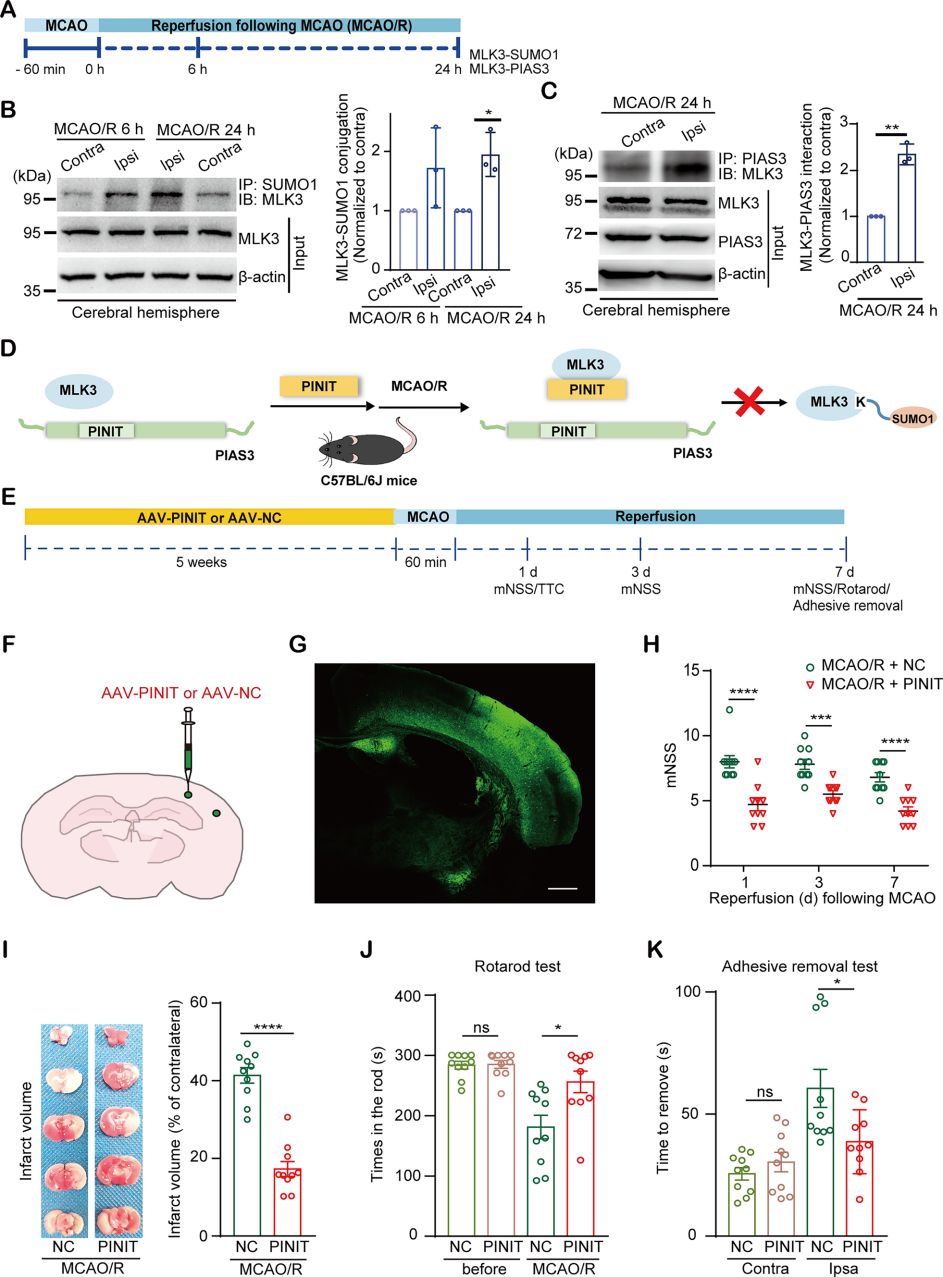
Overexpressing the PINIT domain of PIAS3 reduces brain infarction and rescues motion function defects in mice after focal ischemia. **A** Schematic diagram of the mouse MCAO focal ischemia model. **B**, **C** Changes in MLK3 SUMOylation (**B**) and MLK3-PIAS3 interaction (**C**) in ipsilateral ischemic tissue (Ipsi) and contralateral normal tissue (Contra) in the focal ischemia model. Relative levels were normalized to those of the Contra groups. Data are presented as the mean ± SD (*n* = 3). *P < 0.05; **P < 0.01; paired *t* test. **D**–**E** Schematic diagram of the effect of overexpressing the PINIT domain on PIAS3-mediated MLK3 SUMOylation and reducing neurological defects in a mouse MCAO model. **F** Schematic representation of AAV injection into the mouse right cortex. **G** AAV expression (green) in the mouse cortex five weeks after injection. Bar = 500 μm. **H**–**K** Effects of overexpressing the PINIT domain on mNSS (**H**), brain infarction (**I**), and sensorimotor behaviors assessed by the rotarod test (**J**) and adhesive removal test (**K**). Data are presented as the mean ± SEM (*n* = 10). *P < 0.05; ***P < 0.001; ****P < 0.0001; *ns* nonsignificant. Two-way ANOVA with the Sidak method (**H**) and unpaired *t* test (**I**–**K**)

To determine whether the PINIT domain also has protective effects in focal ischemia, a mouse MCAO model was examined. Similar to the global ischemia model, MLK3-SUMO1 conjugation and MLK3-PIAS3 interaction in the ischemic hemisphere (ipsilateral or Ipsi) increased significantly after MCAO/R compared to those in the nonischemic hemisphere (contralateral or Contra) in the MCAO model (Fig. 7A–C). To determine the effects of the PINIT domain on brain infarction and neurological damage, AAV-PINIT or AAV-NC was stereotactically injected into the cortical regions susceptible to brain injury following MCAO (Fig. 7D–F). Widespread expression was observed five weeks after the injection (Fig. 7G). AAV-PINIT decreased the modified neurological severity score (mNSS) in the MCAO-treated mice after 1, 3, and 7 days of reperfusion compared to that of the AAV-NC group (Fig. 7H). The infarct volume in the ipsilateral cerebral regions was reduced by AAV-PINIT treatment after MCAO/R at 1 (Fig. 7I) and 7 days (Figure S6). Behavioral tests were performed to evaluate the effects of AAV-PINIT on sensorimotor functions in the MCAO-treated mice. In the rotarod test, PINIT overexpression increased the time spent on the rod after MCAO/R, which was not affected before MCAO (Fig. 7J). In the adhesive removal test, compared to the AAV-NC-treated group, the AAV-PINIT group showed a reduced time to remove adhesive tape from the left paw (affected side) and presented no difference in removing tape from the right paw (unaffected side) after MCAO/R (Fig. 7K). These data indicate that the overexpression of the PINIT domain of PIAS3 alleviates neuronal lesions and relieves sensorimotor function deficits in MCAO-treated mice.

## Discussion

Here, the role of MLK3 SUMOylation in ischemic stroke was assessed. Our data identified MLK3 as a target of SUMO1 and provided an additional modification mechanism for regulating MLK3 enzymatic activity. The results showed that MLK3 SUMOylation plays a pivotal role in ischemic neuronal lesions. Blocking the MLK3-PIAS3 interaction inhibits MLK3 SUMOylation and stimulation, alleviates neuronal injury and cognitive impairment, and plays a neuroprotective role after cerebral ischemia.

Disrupted SUMOylation homeostasis was observed in the penumbra tissue of stroke patients, global and focal cerebral ischemic animal models, and in vitro OGD models [32–35]. Some studies suggest that SUMOylation is an endogenous neuroprotective response after cerebral ischemia [38–40, 47]. However, other findings show the opposite. For instance, focal cerebral ischemia increases the expression of SENP1 (sentrin/SUMO-specific protease 1), which then decreases global SUMOylation levels, relieves neural damage, and exerts a protective effect [36]. In addition, SUMOylation of the kainate receptor subunit GluK2 activates the MLK3-JNK3 apoptotic signaling pathway and participates in ischemic neuronal lesions [37]. Based on the above findings, we speculate that cerebral ischemia induces the conjugation of SUMOs with different targets, in which some SUMOylated substrates exert protective effects and others exert damaging effects. Here, we found that MLK3 is a new SUMO1 target and that MLK3 SUMOylation contributes to ischemic cognitive impairment. Clarifying the mechanism for MLK3 SUMOylation will help identify intervention targets and strategies for the treatment of ischemic stroke.

Post-translational modification is recognized as an important way to regulate protein structure and function. Reportedly, MLK3 activity is positively regulated by both autophosphorylation and upstream kinase-mediated phosphorylation at threonine 277 and serine 281 residues within the kinase domain [4], which plays an important role in cell death, contributing to the pathogenesis of ischemic brain damage, Alzheimer’s disease, and oxidative stress injury [10, 37, 48–50]. Here, we found that SUMOylation deficiency at K401 led to decreased phosphorylation of MLK3(T277/S281), revealing crosstalk between SUMOylation and phosphorylation. We deduced that SUMO1 conjugation at K401 increases MLK3(T277/S281) phosphorylation, which in turn potentiates MLK3 catalytic activity. Moreover, we found that SUMOylation stimulates MLK3-p38/JNK apoptotic cascades and ultimately leads to cell apoptosis. Therefore, MLK3 SUMOylation not only participates in ischemic cerebral damage but may also be involved in other pathogenesis mediated by MLK3 activation, such as Alzheimer’s disease and oxidative stress injury.

In SUMOylation cascades, SUMO-activating enzymes (SAE1/2) and SUMO-specific conjugating enzymes (Ubc9) are conserved and unique, whereas SUMO ligases are substrate selective. SUMO ligases include the family of protein inhibitors of activated STAT proteins (PIASs), nuclear pore proteins RanBP2 and Pc2 [23, 24]. The PIASs family is localized in neurons and participates in synaptic plasticity and cognitive function regulation [15, 18, 21, 22, 41, 51–53]. Our results reveal that cerebral ischemia induces MLK3 SUMOylation in the hippocampus and cortex by promoting the interaction of the SUMO ligase PIAS3 and that AAs 133–320 in PIAS3 directly bind MLK3. AAs 133–320 correspond to the PINIT domain of PIAS3, which has 75% homology with PIAS1, 68% homology with PIAS2, and 50% homology with PIAS4. Therefore, whether PIAS1/2/4 plays a role in MLK3 SUMOylation still needs to be elucidated. Moreover, overexpressing the PINIT domain of PIAS3 does not completely block the MLK3-PIAS3 interaction in vivo and in vitro. This finding suggests that other binding regions in PIAS3 cannot be ruled out.

Here, we show that MLK3 SUMOylation leads to MLK3-p38/JNK apoptotic cascade stimulation, neuronal lesions, and cognitive impairment. Overexpression of PIAS3 (133–320) by lentivirus or AAV recombinants interrupted the MLK3-PIAS3 interaction in vivo and in vitro, inhibited MLK3 and downstream apoptotic cascade activation, effectively rescued neuronal lesions, and ameliorated cognitive defects. The PINIT domain (AAs 133–320) of PIAS3 is highly conserved in rats, mice, and humans. Therefore, MLK3-PIAS3 is a potential interruption target for ischemic stroke, and the PINIT peptide is a promising therapeutic biopharmaceutical. Moreover, Tat-PINIT relieves OGD/R-induced dendrite injury and cell apoptosis and presents lower cytotoxicity than Lenti-PINIT. This finding reveals that purified protein may be more suitable for disease treatment. The PINIT domain of PIAS3 contains 188 amino acids, which is a relatively long fragment. Shorter peptides and small molecular compounds need to be identified to be more applicable neuroprotective drugs for ischemic stroke therapy.

In conclusion, our data provide the first evidence that reversible MLK3 SUMOylation is involved in neuronal lesions and cognitive impairment after cerebral ischemia. MLK3-PIAS3 is a potential intervention target for ischemic stroke therapy, and PINIT intervention is a promising neuroprotective strategy.

### Supplementary Information

Below is the link to the electronic supplementary material.

Supplementary file1 (DOCX 3917 KB)

## Data Availability

All data generated or analyzed during this study are included in this published article and its supplementary information files.

## Abbreviations

AAV-NC: AAVs negative control
AAV-PINIT: AAVs expressing the PINIT domain of PIAS3
Co-IP: Co-immunoprecipitation
Contra: Contralateral
His-PIAS3 WT: His-tagged wild-type PIAS3
IB: Immunoblot
IP: Immunoprecipitation
Ipsi: Ipsilateral
I/R: Reperfusion after global ischemia
JNK: C-Jun N-terminal kinase
Lenti-NC: Lentivirus recombinants negative control
Lenti-PINIT: Lentivirus recombinants containing the PINIT domain of PIAS3
MAPKKK: Mitogen-activated protein kinase kinase kinase
MCAO: Middle cerebral artery occlusion
Myc-PIAS3 WT: Myc-tagged wild-type PIAS3
MKKs: MAPK kinases
MLK3 K401R: MLK3 lysine 401 to arginine
MLK3: Mixed-lineage kinase 3
mNSS: Modified Neurological Severity Score
OGD: Oxygen and glucose deprivation
PI: Propidium iodide
SUMOs: Small ubiquitin-like modifiers
Tat-EGFP: Tat-tagged EGFP
Tat-PINIT: Tat-tagged PINIT domain of PIAS3
