# Supplementary material for: Disrupting PIAS3-mediated SUMOylation of MLK3 ameliorates poststroke neuronal damage and deficits in cognitive and sensorimotor behaviors
Source: Cell Mol Life Sci. 2024 Mar 8;81(1):119. doi: 10.1007/s00018-024-05166-7 (PMC10924033; doi:10.1007/s00018-024-05166-7)
Supplement: Supplementary file 1 — Supplementary file1 (DOCX 3917 KB) [file 18_2024_5166_MOESM1_ESM.docx]

**Supplement Figures and captions**

**
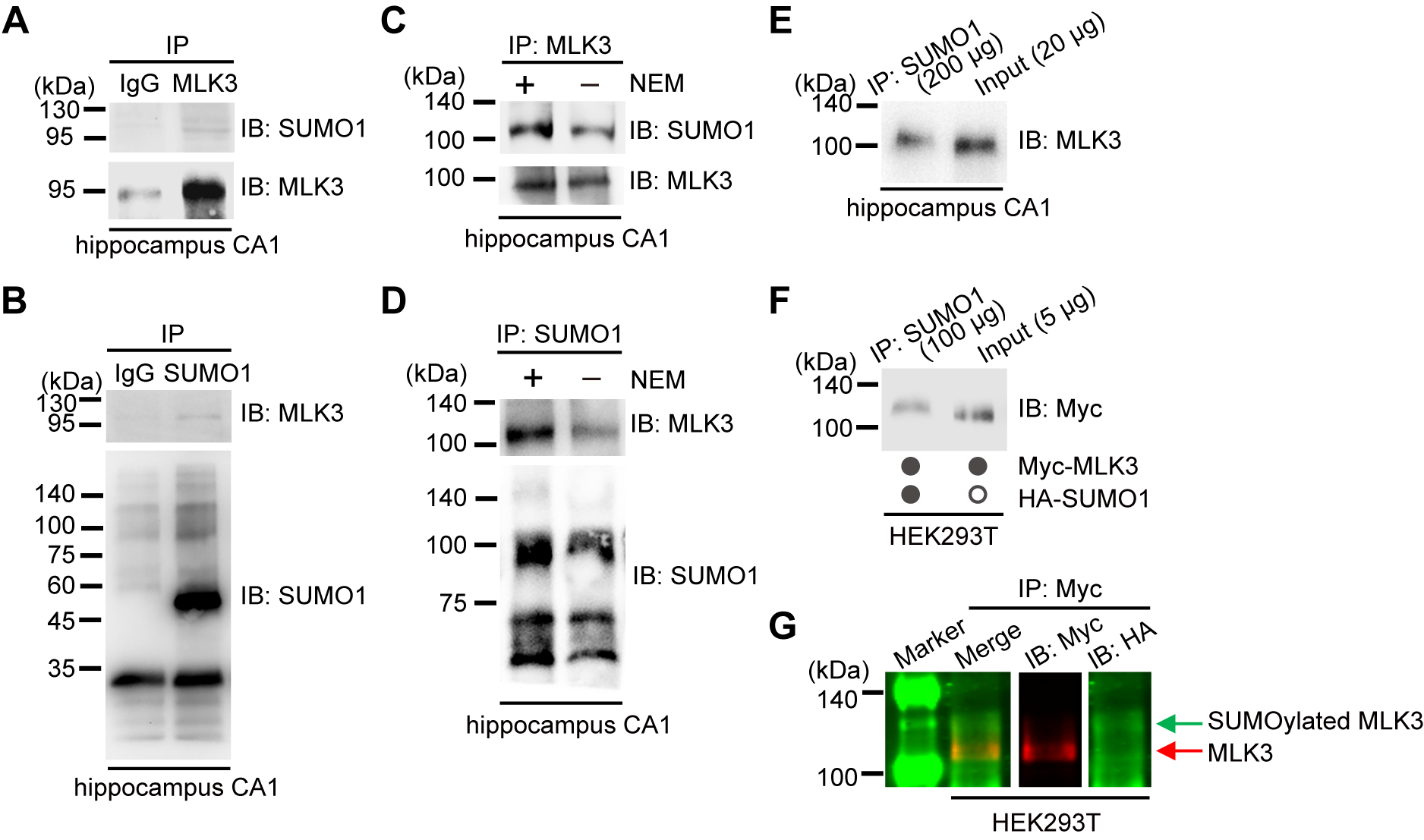
**

**Supplement Figure 1. MLK3 is conjugated with SUMO1.** (A-E) MLK3 was modified by SUMO1 in rat hippocampus CA1 subfield. (A) Co-IP with MLK3 antibody or IgG and immunoblot with SUMO1 antibody. (B) Co-IP with SUMO1 antibody or IgG and immunoblot with MLK3 antibody. IgG, nonspecific IgG. (C,D) Detection of MLK3 SUMOylation with (+) or without (-) N-ethylmaleimide (NEM) by co-IP. NEM was used to inhibit *in vitro* protein deSUMOylation. (E) Detection of MLK3-SUMO1 interaction by ECL method. (F-G) MLK3 was modified by SUMO1 in HEK293T cells. HEK293T cells were cotransfected with indicated expression plasmids, and then subjected to Co-IP. (F) Detection of MLK3 SUMOylation by ECL method. Input, HEK293T cells overexpressing Myc-MLK3. (G) Detection of MLK3 SUMOylation by dual infrared laser imaging system. HEK293T cells overexpressing Myc-MLK3 and HA-SUMO1 were subjected to co-IP with Myc antibody followed by immunoblotting with HA (green) and Myc (red) antibody together. The green band represents the SUMO-1ylated MLK3, and the red band represents unmodified MLK3.


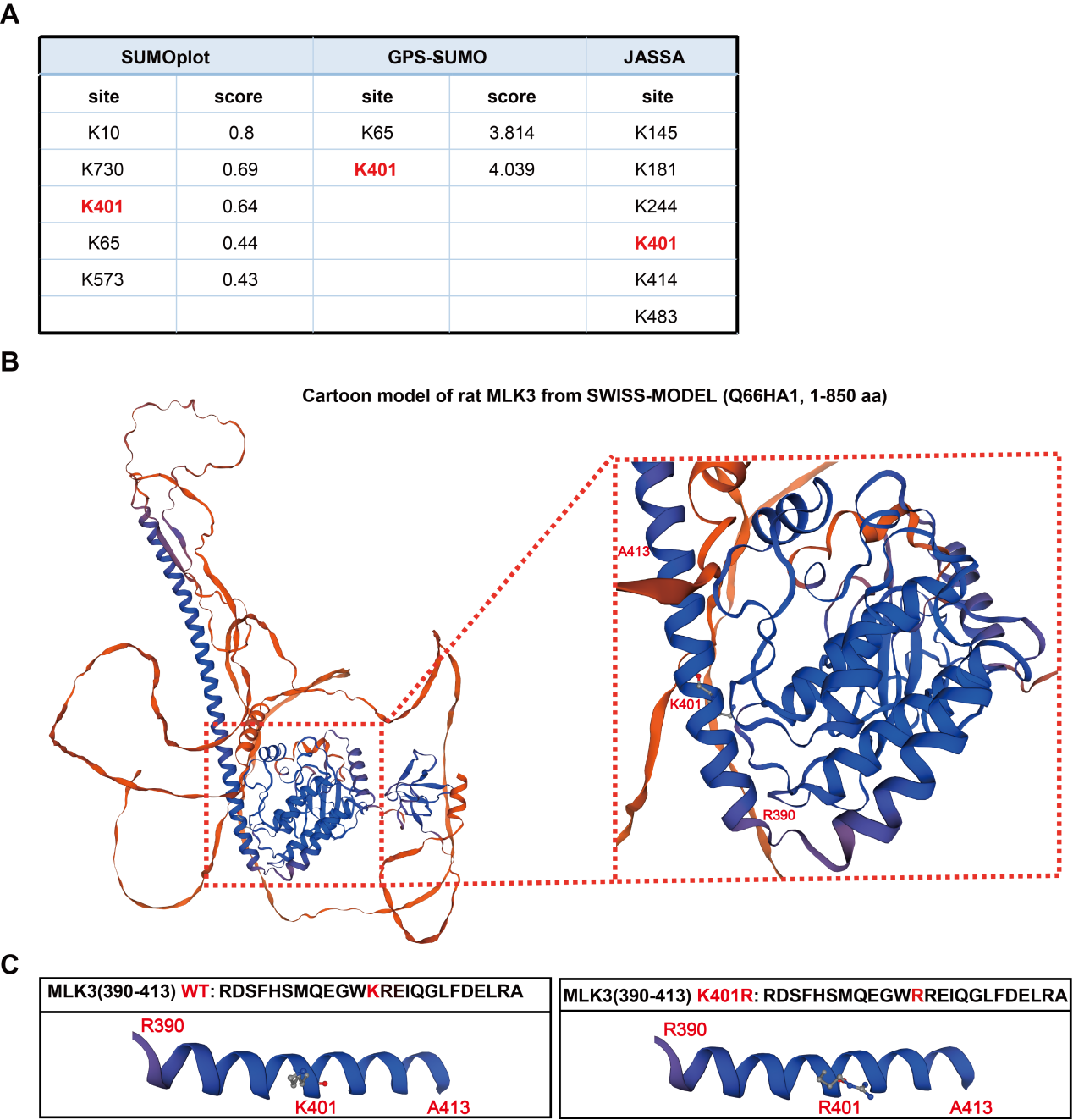


**Supplement Figure 2. Prediction of SUMOylation sites of MLK3 and three-dimensional structure of MLK3.** (A) Predication of SUMOylated sites in MLK3. Common predicted K401 site was highlighted in red. (B) Cartoon model of rat MLK3 from SWISS-MODEL (Q66HA1, 1-850 aa). (C) Three-dimensional structure of amino acids 390-413 of MLK3 [MLK3(390-413)] and its K401R mutant from SWISS-MODEL.


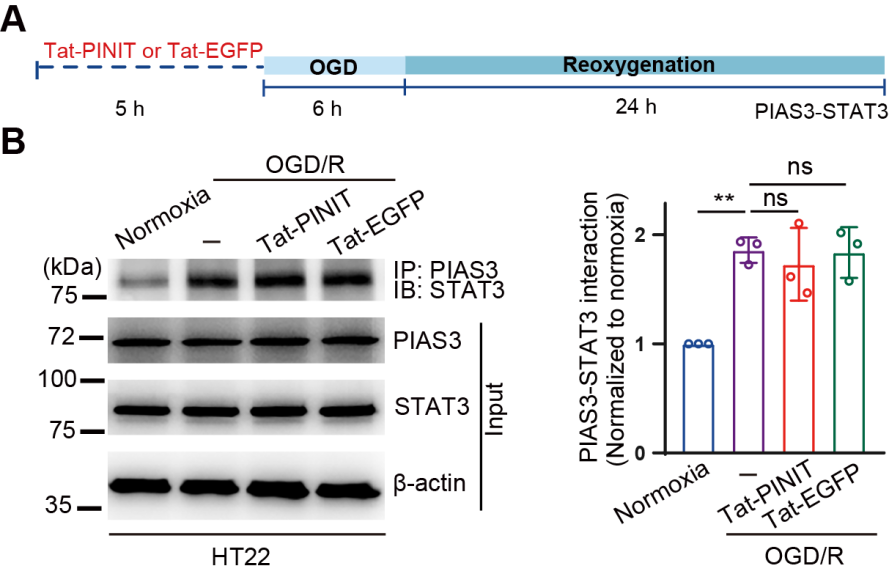


**Supplement Figure 3. Tat-PINIT does not significantly affect OGD/R-induced STAT3-PIAS3 interaction.** (A) Schematic diagram of the effect of Tat-PINIT or Tat-EGFP (negative control) on STAT3-PIAS3 interaction in the OGD model of HT22 cells. (B) The effects of overexpressing the PINIT domain (5 μmol/L) on OGD/R-induced STAT3-PIAS3 interaction by co-IP and immunoblot analysis. −, without Tat treatment. Relative levels were normalized to the normoxia. Data are presented as the mean ± SD (n=3). ***P*<0.01; ns, nonsignificant; one-way ANOVA with Dunnett’s *t* test.


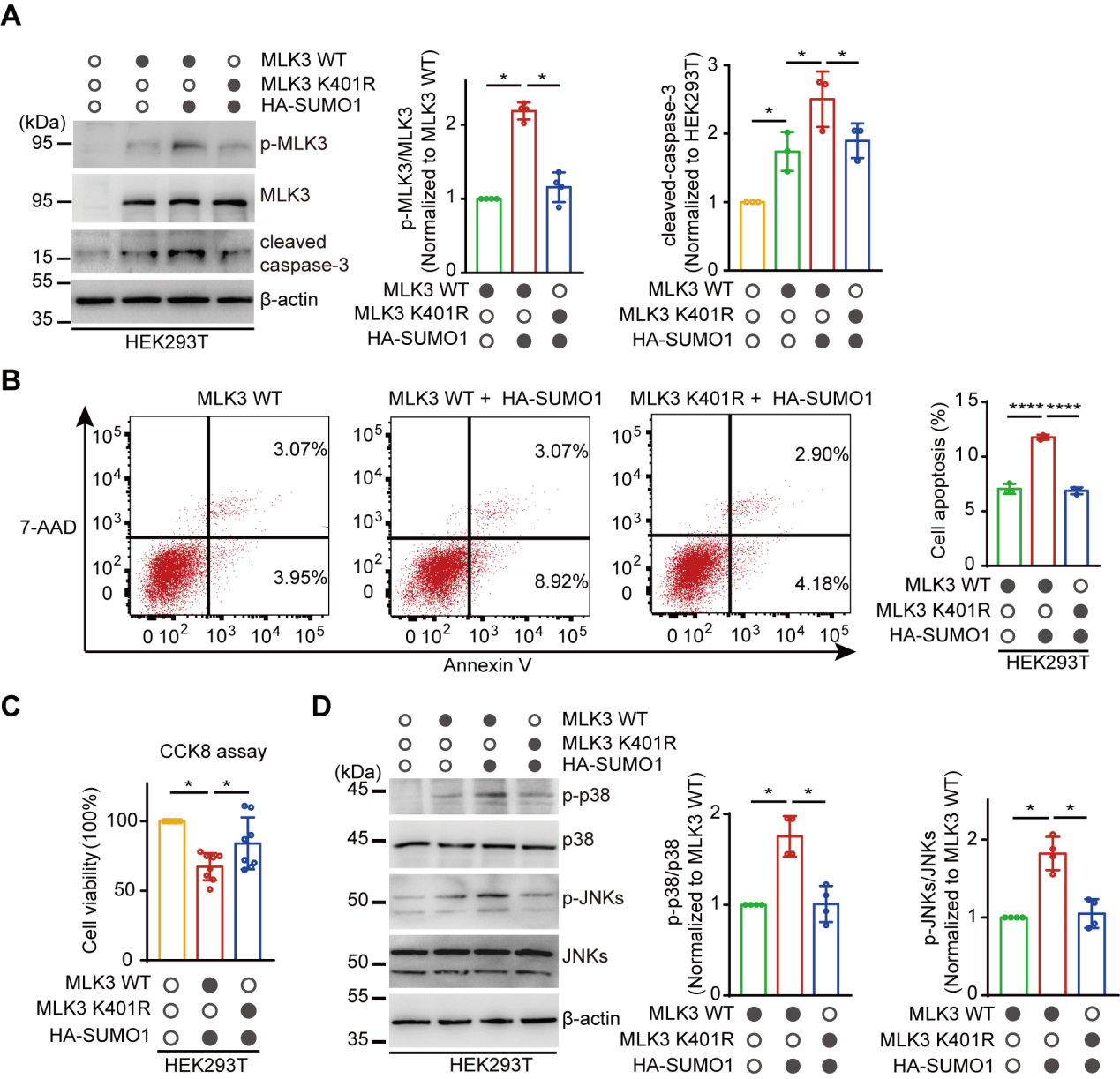


**Supplement Figure 4. MLK3_-_K401 SUMOylation stimulates MLK3-JNKs/p38 signaling cascades and leads to cell apoptosis.** HEK293T cells were co-transfected with plasmids expressing HA-SUMO1 and MLK3 (WT or K401R), and then assessed by immunoblot (A, D), flow cytometric apoptotic analysis (B), and CCK-8 assay (C). Relative levels were normalized to MLK3 WT transfection group (A, C, D). Data are presented as mean ± SD (n=3-8). **P*<0.05; *****P*<0.0001; one-way ANOVA with Dunnett’s *t* test.


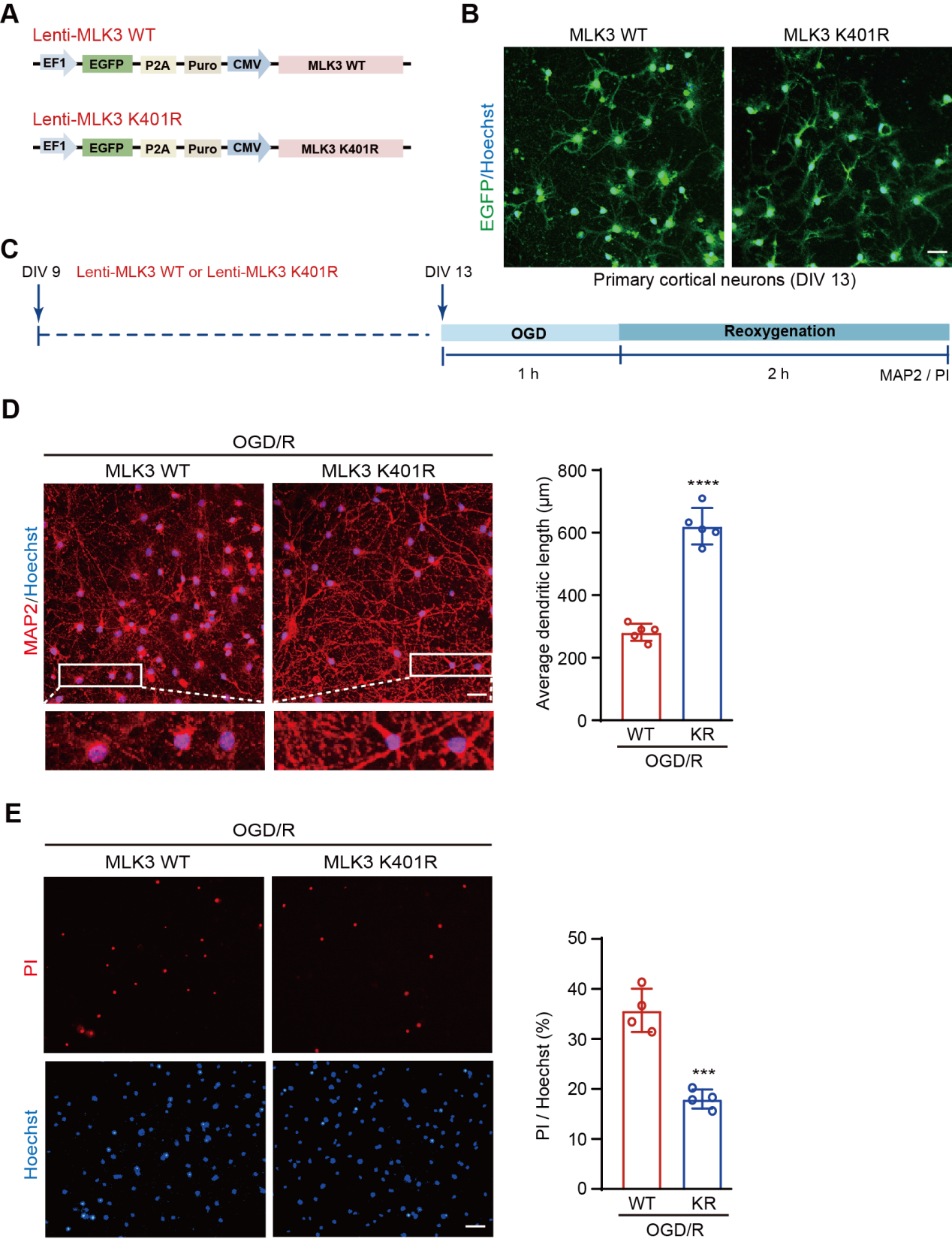


**Supplement Figure 5. MLK3 K401R mutation alleviates neuronal injury after OGD/R.** (A) Diagram of lentiviral plasmids expressing MLK3 WT and K401R mutant. (B) Lentivirus infection efficiency (green) in primary cortical neurons. Bar = 50 μm. (C) Schematic diagram of the overexpression of the MLK3 WT and K401R mutant in the primary neuron OGD model. (D, E) The effects of overexpressing the MLK3 WT and K401R mutant on OGD/R-induced neuronal dendrite injury (D) and cell apoptosis (E). The average length of dendrites is presented as the total dendritic length (MAP2) divided by the number of cells. Bar = 50 μm. Data are presented as the mean ± SD (n=4-5). ****P*<0.001; *****P*<0.0001; unpaired *t* test.


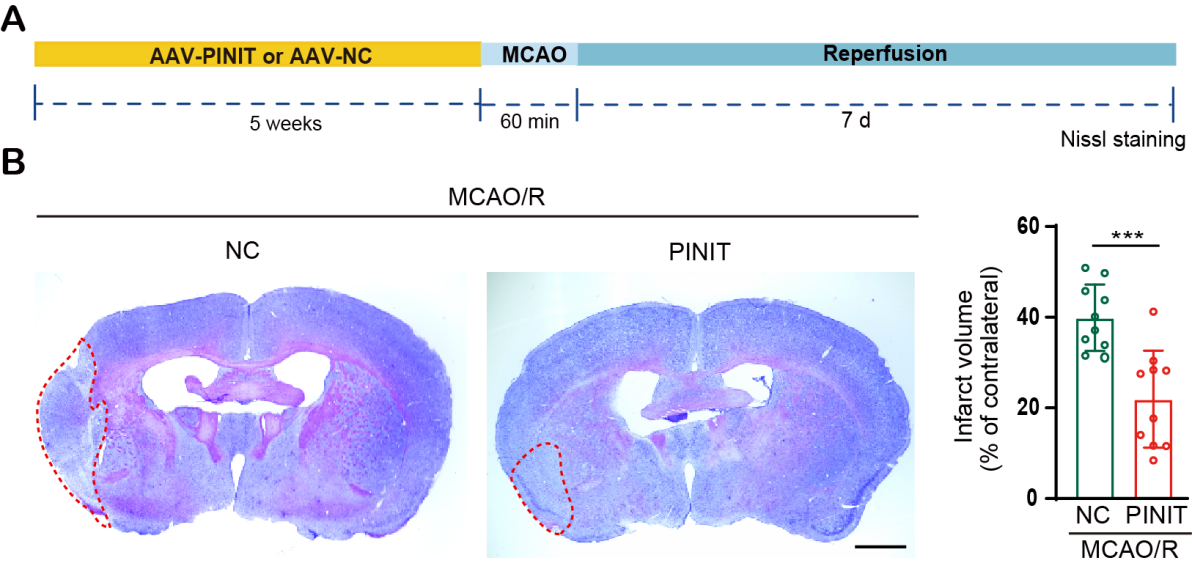


**Supplement Figure 6. Overexpressing the PINIT domain of PIAS3 reduces brain infarction in mice after MCAO/R 7 d.** (A) Schematic diagram of the effect of overexpressing the PINIT domain in a mouse MCAO model. (B) Effects of overexpressing the PINIT domain on brain infarction detected by Nissl staining. Bar = 1 mm. Data are presented as the mean ± SEM (n=10). ****P*<0.001; unpaired *t* test.
